# Sphingosine-1-phosphate/S1P Receptors Signaling Modulates Cell Migration in Human Bone Marrow-Derived Mesenchymal Stem Cells

**DOI:** 10.1155/2014/565369

**Published:** 2014-07-23

**Authors:** Yaxian Kong, Hong Wang, Tao Lin, Shuling Wang

**Affiliations:** ^1^Institute of Infectious Diseases, Beijing Ditan Hospital, Capital Medical University, Beijing 100015, China; ^2^Beijing Key Laboratory of Emerging Infectious Diseases, Jingshundongjie 8, Beijing 100015, China; ^3^Stem Cell Research Center, Peking University, Beijing 100191, China

## Abstract

The recruitment of bone marrow-derived mesenchymal stem cells (BMSCs) to damaged tissues and sites of inflammation is an essential step for clinical therapy. However, the signals regulating the motility of these cells are still not fully understood. Sphingosine-1-phosphate (S1P), a bioactive sphingolipid metabolite, is known to have a variety of biological effects on various cells. Here, we investigated the roles of S1P and S1P receptors (S1PRs) in migration of human BMSCs. We found that S1P exerted a powerful migratory action on human BMSCs. Moreover, by employing RNA interference technology and pharmacological tools, we demonstrated that S1PR1 and S1PR3 are responsible for S1P-induced migration of human BMSCs. In contrast, S1PR2 mediates the inhibition of migration. Additionally, we explored the downstream signaling pathway of the S1P/S1PRs axis and found that activation of S1PR1 or S1PR3 increased migration of human BMSCs through a G_*i*_/extracellular regulated protein kinases 1/2- (ERK1/2-) dependent pathway, whereas activation of S1PR2 decreased migration through the Rho/Rho-associated protein kinase (ROCK) pathway. In conclusion, we reveal that the S1P/S1PRs signaling axis regulates the migration of human BMSCs via a dual-directional mechanism. Thus, selective modulation of S1PR's activity on human BMSCs may provide an effective approach to immunotherapy or tissue regeneration.

## 1. Introduction

Mesenchymal stem cells (MSCs) have been shown to affect both innate and adaptive immune response [1–3]. They have been identified and isolated from multiple tissues, including adipose tissue, umbilical cord, bone marrow, muscle, and fetal liver [4]. Increasing evidence suggests that bone marrow-derived mesenchymal stem cells (BMSCs) have therapeutic potential due to their immunosuppressive property in many immunological disorders, including graft-versus-host disease [5], Crohn's disease [6], and the prevention of organ transplantation rejection [7, 8]. Furthermore, many studies have demonstrated that BMSCs play a critical role in injury healing. BMSC transplantation is also regarded as a useful therapeutic strategy in acute tissue injuries of the lung, heart, liver, and kidney [9–12]. Of note, in all of these preclinical and clinical studies, the engraftment of BMSCs into damaged tissues via migration to suppress immune responses or enhance tissue repair/regeneration is a crucial process for clinical therapy [13]. For BMSCs to migrate to and target a specific tissue, they require the right combination of signaling molecules from the injured tissue and the corresponding receptors on BMSCs [13, 14].

Among the chemokines and inflammatory mediators known to trigger potent cellular chemotaxis, sphingosine-1-phosphate (S1P), which is a bioactive sphingolipid metabolite, is an ideal candidate [15]. S1P is reported to have a variety of biological effects on cells, including modulation of motility, proliferation, differentiation, apoptosis, survival, neurite retraction, angiogenesis, and regulation of immune function [16, 17]. S1P can act as both an intracellular second messenger and a ligand for a family of G protein-coupled receptors referred to as S1P receptor types 1–5 (S1P1–5) [18]. The distinct response of each cell line to S1P varies depending on its S1PR expression pattern. S1PR1, S1PR2, and S1PR3 specifically contribute to S1P-induced cell motility [15]. Previous studies by us and other researchers have demonstrated that S1P strongly stimulated BM cells migration and induced morphological rearrangements in mice [19–21]. However, the effects of S1P signaling on human BMSCs and the mechanisms that regulate their chemotactic behavior are incompletely understood.

In the present study, we characterized the different effects of S1P receptors on S1P-mediated migration of human BMSCs and investigated the downstream signaling pathway in this process. Understanding the role of S1P/S1PRs on human BMSC migration will promote their effective use for immunotherapy or tissue regeneration.

## 2. Material and Methods

### 2.1. Chemicals and Reagents

Minimum essential medium *α* (MEM *α*), penicillin, streptomycin, L-glutamine, and trypsin were purchased from GIBCO (Grand Island, NY, USA). Fetal bovine serum was from Biochrom (Berlin, Germany). Fluorescence-conjugated monoclonal antibodies for CD44, CD105, CD166, CD73, CD14, CD34, and CD45 were from BD Pharmingen (San Diego, CA, USA). Rabbit polyclonal antibodies to S1PR1–3, *α*-tubulin, phosphorylated-extracellular regulated protein kinases 1/2 (p-ERK1/2), and total ERK1/2 were from Santa Cruz Biotechnology (San Diego, CA, USA). PCR reagents and probes used for real-time PCR were from Applied Biosystems (Foster City, CA, USA). S1P and dihydro-S1P (H_2_S1P) were from Biomol (Tebu, France). S1PR1 agonist (SEW2871), S1PR1 antagonist (W146), S1PR2 antagonist (JTE-013), and S1PR3 antagonist (CAY10444) were from Cayman Chemical (Ann Arbor, MI, USA). MEK inhibitor U0126 and Rho kinase inhibitor Y27632 were from Calbiochem (San Diego, CA, USA). Bovine serum albumin (BSA) and other common reagents were from Sigma (St. Louis, MO, USA).

### 2.2. Isolation of Human BMSCs and Peripheral Blood Mononuclear Cells (PBMCs)

Human BMSCs were obtained from three healthy donors as described previously [22]. Briefly, bone marrow cells were resuspended in modified Eagle's medium of alpha containing 10% fetal bovine serum, 100 U/mL penicillin, 100 *μ*g/mL streptomycin, and 2 mM L-glutamine and plated in a flask at a density of 3 × 10^5^ cells/mL. Nonadherent cells were discarded after cultivation for 48 h. The adherent cells were washed twice and cultured for 10 to 14 days until cell clones formed. Venous blood was obtained from three healthy donors and was collected directly into BD-Vacutainer CPT tubes (BD Biosciences). PBMCs were isolated by centrifugation according to the manufacturer's instructions. The viability of PBMCs was determined by trypan blue staining and was found to be >90%. Permissions to use human tissue and blood were granted by Ethical Committee of Peking University Health Science Center and Beijing Ditan Hospital, Capital Medical University.

### 2.3. Flow Cytometry Analysis

The surface markers of human BMSCs were analyzed by flow cytometry. Cells were harvested by trypsinization, washed once with PBS, and resuspended in PBS containing 2% FBS. The cells were incubated with the conjugated antibodies for 30 min on ice. The following antibodies against human antigens were used: phycoerythrin- (PE-) conjugated anti-CD34, anti-CD45, anti-CD73, anti-CD105, and anti-CD166 and fluorescein isothiocyanate- (FITC-) conjugated anti-CD14 and anti-CD44. Corresponding isotype-matched control monoclonal antibodies were used in all flow cytometric staining procedures. Flow cytometric analysis was performed using a FACS Calibur (BD Biosciences, San Diego, CA, USA).

### 2.4. Western Blot Analysis

To monitor the activation of ERK1/2, cells were exposed to S1P (1 *μ*M) for various times (2–120 min). Where indicated, cells were pretreated with S1PR antagonists for 1 h before S1P stimulation (1 *μ*M, 2 min). Cells were lysed in lysis buffer (50 mM Tris-Cl pH 8.0, 150 mM NaCl, 1% Nonidet P-40, 0.1% sodium dodecyl sulfate (SDS), 1% Triton X-100, 1 mM sodium orthovanadate, 1 mM phenylmethylsulfonyl fluoride (PMSF), and 10 *μ*g/mL aprotinin/leupeptin) containing protease inhibitors. Total protein concentration was determined by Bradford assay. Equal amounts (30 mg) of total protein were separated by 10% SDS-polyacrylamide gels (SDS-PAGE). For analysis of S1PR1-3 expression, cells were lysed in lysis buffer as described before. Protein samples (100 mg) were separated by 10% SDS-PAGE. Proteins were later transferred from polyacrylamide gel to methanol-soaked Immobilon polyvinylidene difluoride membranes (Millipore, Bedford, MA). Primary antibody incubation was performed overnight at 4°C. The membranes were then washed three times and incubated with appropriate HRP-conjugated secondary antibodies (1 : 1000, Santa Cruz) at room temperature for 1 h. The membranes were then washed three times and the signals were visualized using an enhanced chemiluminescence (ECL Plus) assay kit (Perkin Elmer, Boston, MA, USA). The bands were quantified using GeneSnap software (Perkin Elmer).

### 2.5. Migration Assay

*In vitro* migration was evaluated using a transwell chamber assay (Millipore, Billerica, MA, USA) as previously described [21]. In brief, BMSCs were serum-starved overnight. Where indicated, cells were transfected with S1PR1-3 siRNA 48 h prior to S1P stimulation (1 *μ*M, 5 min) or pretreated for 1 h with W146 (S1PR1 antagonist), JTE-013 (S1PR2 antagonist) or CAY10444 (S1PR3 antagonist). Then, 4 × 10^4^ cells were seeded to the upper chamber. Various concentrations of S1P, H_2_S1P, or SEW2871 (S1PR1 agonist) were added to the lower chamber. The chambers were incubated for 4 h at 37°C in 5% CO_2_. Migrated cells, which adhered to the lower face of the porous membrane, were fixed with methanol at 4°C for 1 h and stained with hematein staining solution. Unmigrated cells on the upper membrane surface were removed with a cotton swab. The migration was quantified by analyzing at least six random fields per filter for each independent experiment.

### 2.6. Real-Time PCR

Total RNA was extracted from cells using the RNeasy kit (Qiagen, Hilden, Germany). cDNA was synthesized from the total RNA sample using High Capacity cDNA Reverse Transcription Kits (Applied Biosystems). Real-time PCR was performed in an ABI Prism 7500 sequence detection system (Applied Biosystems). The expression of the gene of interest was calculated relative to the levels of GAPDH mRNA (Δct). The expression levels are presented as “fold change” relative to values obtained with the control (set as “onefold”).

### 2.7. RNA Interference

The siRNA sequence targeting specifically human S1PR1–3 was synthesized by Ambion (Grand Island, NY, USA). BMSCs at 40–50% confluency were prepared. Transient transfection of siRNA (40 nM) was performed using Lipofectamine RNAiMAX (Invitrogen, Carlsbad, CA, USA) as recommended by the manufacturer. Control cells were treated with 40 nM RNAi Negative Control Duplexes (scramble siRNA). After 48 h, the transfected cells were used to perform migration assays.

### 2.8. RhoA Activation Assay

RhoA activation was assessed using the Rho Assay Reagent (Millipore) according to the manufacturer's procedure and as described previously [19]. Briefly, human BMSCs were grown to approximately 80% confluence on 100 mm dishes and serum-starved in MEM *α* for 12 h and then treated with or without the above-described S1PRs antagonists before they were stimulated with 1 *μ*M S1P. Immediately after stimulation for the indicated time, cells were rinsed with cold PBS, lysed in 300 *μ*L of lysis buffer (25 mM HEPES, pH 7.5, 150 mM NaCl, 1% NP-40, 10 mM MgCl_2_, 1 mM EDTA, 2% glycerol, 25 mM NaF, 1 mM sodium orthovanadate, 1 mM phenylmethylsulfonyl fluoride (PMSF), and 10 *μ*g/mL aprotinin/leupeptin), and then briefly centrifuged to remove cell debris. Cell lysates were pulled down by incubation at 4°C with 20 *μ*g of recombinant Rhotekin-binding domain bound to glutathione-agarose beads for 1 h. Following washing, bound Rho was eluted by SDS sample buffer. The eluted samples and the total cell lysates were then subjected to western blot analysis with RhoA antibody to detect active and total RhoA, respectively.

### 2.9. Statistical Analysis

Data are expressed as means ± SD and were analyzed by Student's* t*-test when appropriate. *P* < 0.05 was considered statistically significant.

## 3. Results

### 3.1. Human Bone Marrow-Derived Stem Cells Express S1PR1, S1PR2, and S1PR3

**Figure 1:**
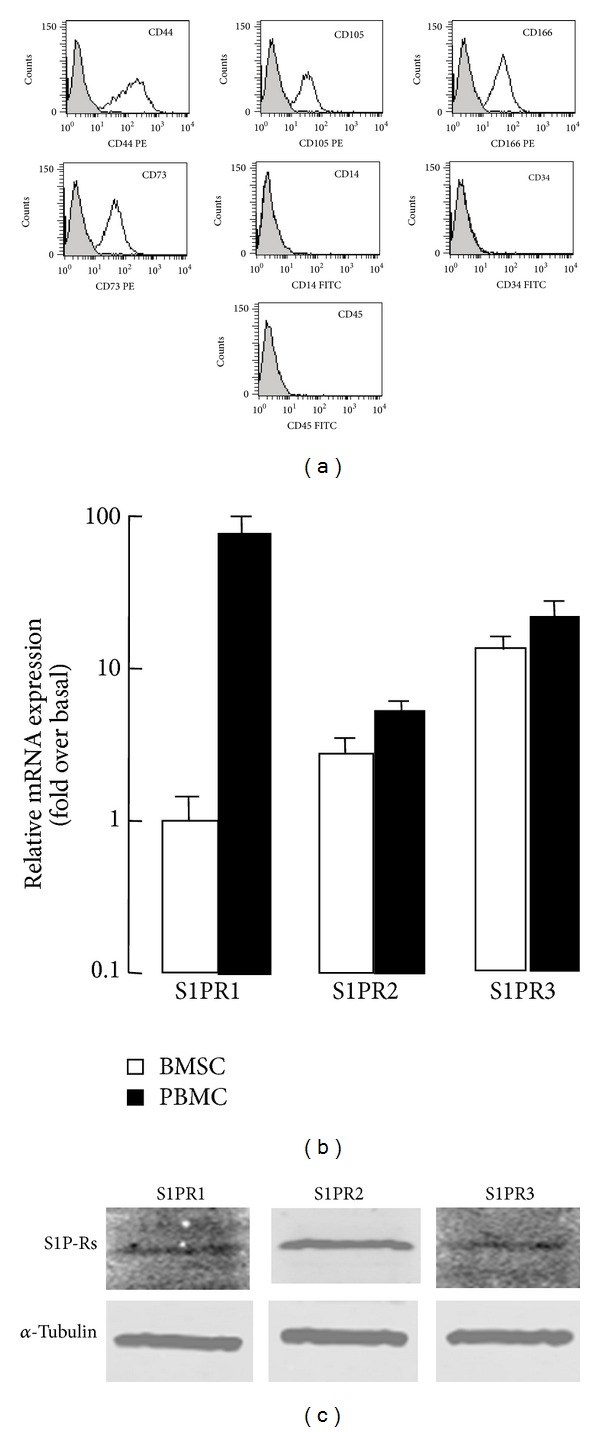
Expression of S1PRs in BMSCs. (a) The identification of BMSCs was performed by flow cytometry analysis. (b) Real-time PCR analysis for expression of S1PR1–3 in BMSCs. Human PBMCs as a positive control. (c) Western blot analysis for expression of S1PR1–3 in BMSCs.

In line with previous studies, human BMSCs were confirmed to express CD44, CD105, CD166, CD73, and lack expression of CD14, CD45, and CD34 (Figure 1(a)). Since S1PR1–3 are the S1P cell surface receptor subtypes that are specifically involved in S1P-mediated biological activities; we investigated whether these receptors are expressed in human BMSCs. Real-time PCR and western blot analysis indicated that these receptors were detectable in human BMSCs in mRNA and protein level (Figures 1(b) and 1(c)).

### 3.2. S1P Induces Human BMSC Migration through Cell Surface Receptors

**Figure 2:**
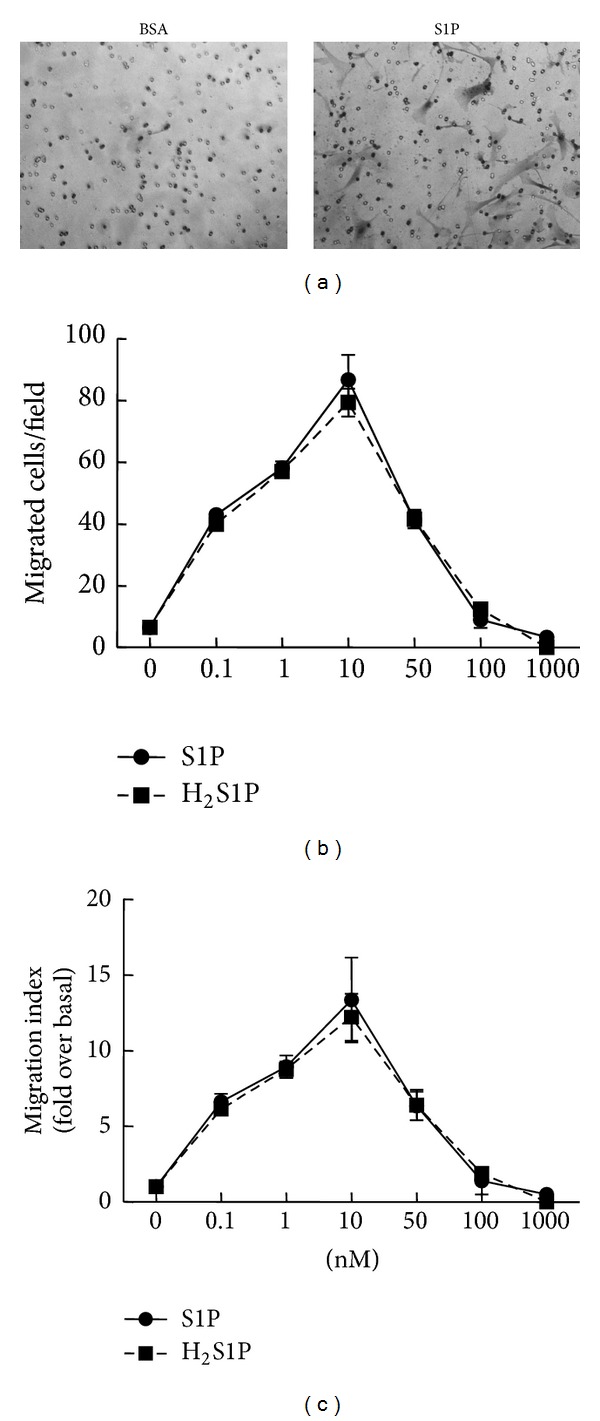
S1P-induced migration of human BMSCs via cell surface receptor. (a) The representative images of serum-starved BMSC migration stimulated with BSA or 1 nM S1P for 4 h. (b)-(c) Serum-starved BMSCs were allowed to migrate for 4 h in the presence of varying concentrations of S1P and H_2_S1P, as indicated. Migrated cells in a random fields (b) or migration index (fold over basal, (c)) shown were counted in 10 random fields per filter for each condition. Data are presented as the mean ± SD. *P < 0.05, compared with control.

To investigate the chemotaxis of human BMSCs in response to various concentrations of S1P, we used the transwell assay and found that low concentrations of S1P (1–10 nM) exerted a strong dose-dependent migration effect (Figures 2(a)–2(c)). Meanwhile, higher concentrations of S1P were less effective and even inhibitory (Figures 2(b) and 2(c)).

Since S1P can act as both an intracellular second messenger and a ligand for a family of G protein-coupled receptors, it was of interest to test whether S1P triggers the migration of human BMSCs via the receptors or not. Therefore, we performed the same experiments using the structural analogue of S1P, H_2_S1P, which is only able to mediate its effects through surface-bound S1PRs [23]. As expected, H_2_S1P completely mimicked the induced migration activity of S1P on human BMSCs (Figure 2(b)), which suggested that S1P induced these actions via activation of membrane S1PRs.

### 3.3. S1PR1 and S1PR3 Mediate Promotion of Migration in Human BMSCs

**Figure 3:**
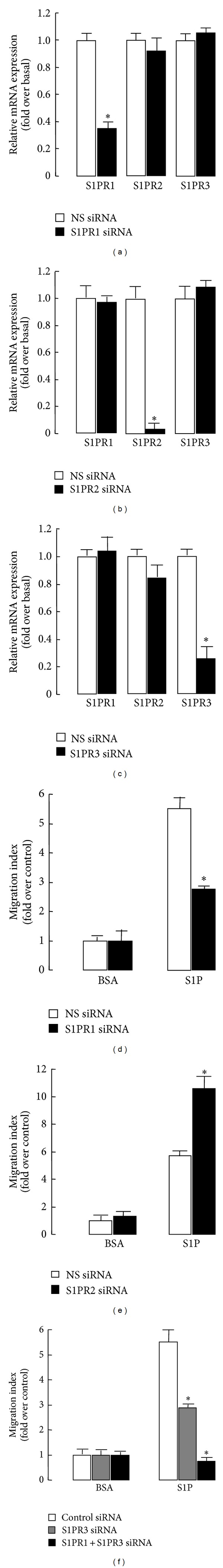
The effect of silencing S1PR expression on S1P-induced migration in human BMSCs. (a)–(c) Cells were transfected with control siRNA or with siRNA targeted against S1PR1 (a), S1PR2 (b), or S1PR3 (c) for 48 h. Then S1PR1, S1PR2, or S1PR3 mRNA was evaluated by real-time RT-PCR. (d)–(f) Effect of silencing S1PR1, S1PR2, or S1PR3 expression on human BMSCs migration in response to S1P. Data are presented as the mean ± SD. *P < 0.05, compared with control siRNA.

S1P has been reported to either promote or inhibit cellular migration, depending on the cell type examined, via different receptors. Therefore, a series of techniques were employed to explore the unique effects of S1P receptors on the migration of human BMSCs. First, we used siRNA technology to knock down S1PR1 and S1PR3 expression in human BMSCs. To validate this approach, the mRNA levels of S1PR1 and S1PR3 in cells treated with siRNA were measured at 48 h after transfection. Human BMSCs transfected with siRNA targeting S1PR1 or S1PR3 showed a marked reduction in S1PR1 or S1PR3, whereas the two siRNAs did not alter the expression of other S1PRs, which confirmed their specificity (Figures 3(a) and 3(c)). Silencing of S1PR1 or S1PR3 expression by siRNA effectively attenuated the migratory effect induced by S1P (Figures 3(d) and 3(f)). Moreover, transfection with a combination of S1PR1 and S1PR3 siRNA completely abrogated S1P-mediated migration (Figure 3(f)).

**Figure 4:**
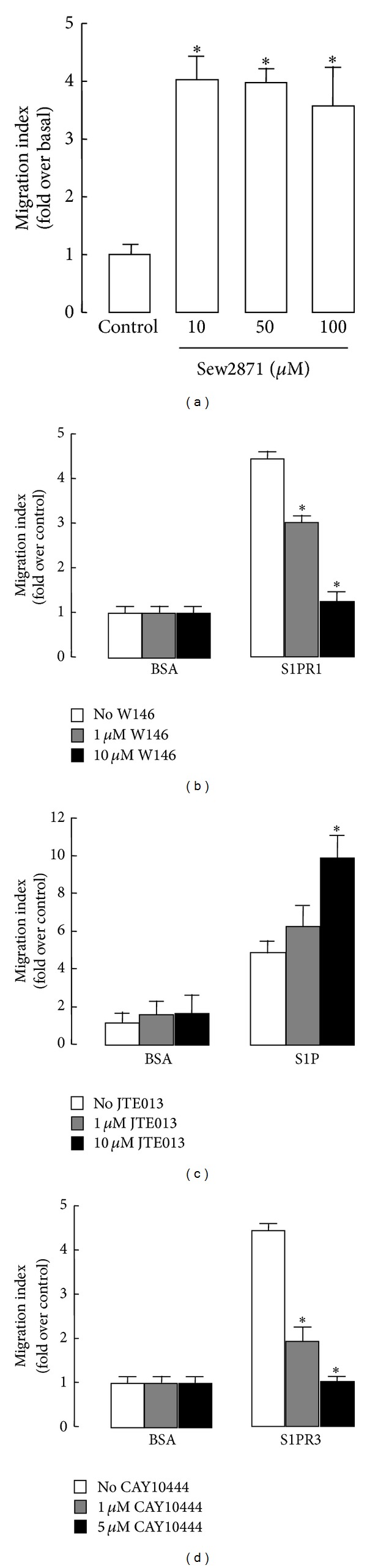
The effect of S1PRs agonist or antagonist on S1P-induced human BMSCs migration. (a) Serum-starved cells were allowed to migrate for 4 h in the presence of the indicated concentration of SEW2871, an S1PR1 agonist. (b)–(d) Serum-starved cells were pretreated for 1 h with or without the S1PR1 antagonist W146 (b), S1PR2 antagonist JTE-013 (c), and S1PR3 antagonist CAY10444 (d). Pretreated cells were then allowed to migrate in the presence of S1P. Data are presented as the mean ± SD. *P < 0.05, compared with control.

To verify this notion, selective S1PRs agonists and/or antagonists were employed. Human BMSCs displayed a marked migratory response towards SEW2871, a selective S1PR1 agonist, in a dose-dependent manner (Figure 4(a)). Moreover, S1P-induced migration of human BMSCs was completely abrogated by the S1PR1 antagonist W146 or the S1PR3 antagonist CAY10444, in a dose-dependent manner (Figures 4(b) and 4(d)). These results indicated that S1PR1 and S1PR3 both contributed to the S1P-induced migration of human BMSCs.

### 3.4. S1PR2 Mediates Inhibition of Migration in Human BMSCs

We also used siRNA and pharmacological reagent to evaluate the effect of S1PR2 on cell migration. The reduced expression of S1PR2 mRNA in cells treated with S1PR2 siRNA validated the approach (Figure 3(b)). The siRNA against S1PR2 and the S1PR2 antagonist JTE-013 both potently enhanced the S1P-induced migration of BMSCs (Figures 3(e) and 4(c)). In agreement with many previous reports, these data demonstrate that S1PR2 negatively regulated migration mediated by S1P in human BMSCs.

Taken together, these results demonstrate that S1PR1 and S1PR3, but not S1PR2, are responsible for S1P-induced migration of human BMSCs.

### 3.5. S1P-Induced Migration Is G_*i*_ Dependent, and the ERK1/2 Pathway Is Involved in This Process

**Figure 5:**
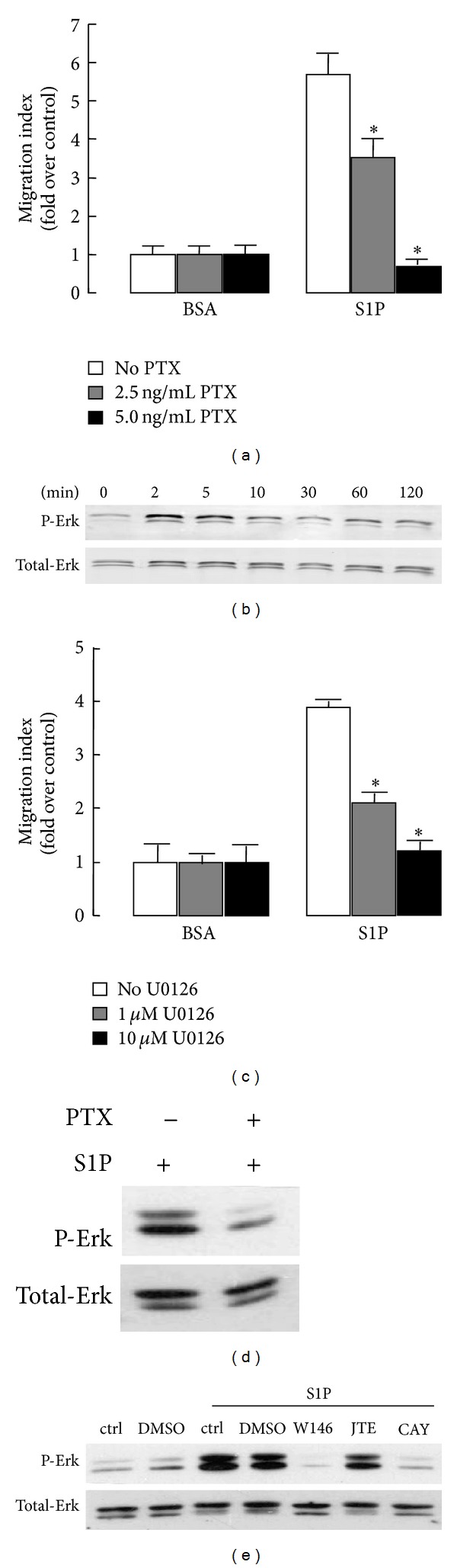
Involvement of the PTX-sensitive ERK1/2 pathway in S1P-induced migration of human BMSCs. (a), (c) Serum-starved BMSCs were pretreated with the indicated concentration of PTX (a) or U0126 (c) and then were allowed to migrate for 4 h in the presence of 1 nM S1P. (b) The effect of S1P on the activation of ERK1/2 MAPK. Cells were stimulated with S1P (1 *μ*M) for the indicated time and cell lysates were analyzed by western blot. (d)-(e): The effect of S1PRs antagonists (S1PR1 antagonist W146, S1PR2 antagonist JTE-013, and S1PR3 antagonist CAY1444) and PTX on S1P-induced activation of ERK1/2. Cells were pretreated with S1PRs antagonists (d) or pretreated with 5 ng/mL PTX (e). The pretreated cells were stimulated with 1 *μ*M S1P for 2 min, and cell lysates were analyzed by western blot. Data are presented as the mean ± SD. *P < 0.05, compared with control.

Since receptors for S1P are coupled to PTX-sensitive G_*i*_ as well as PTX-insensitive G proteins such as G_*q*_ and/or G_12/13_ proteins [24], we performed experiments with cells pretreated with PTX. PTX completely blocked migration mediated by S1P (Figure 5(a)), which suggested that S1P-induced migration in human BMSCs was G_*i*_ protein dependent.

Signaling through ERK1/2 activation has been shown to mediate S1P-induced migration [19, 25, 26]. Indeed, we found that S1P induced rapid and transient phosphorylation of ERK1/2 in a time-dependent manner (Figure 5(b)), indicating the activation of the ERK1/2 pathway. Treatment of these cells with U0126, a specific inhibitor of ERK1/2 phosphorylation, strongly blocked S1P-mediated migration (Figure 5(c)). Previous studies reported the involvement of PTX-sensitive G_*i*_ proteins in S1P receptors signal transduction [23, 24] and that *β* *γ* subunits from G_*i*_ proteins can induce activation of ERK1/2 [27]. This prompted us to investigate the effect of PTX on S1P-induced ERK1/2 activation in human BMSCs. As shown in Figure 5(d), pretreatment with PTX blocked the activation of ERK.

Furthermore, the S1PR1 is known to couple to G_*i*_, whereas S1PR2 and S1PR3 couple to G_12/13_, G_*q*_, and G_*i*_ [24]. Thus, to investigate whether blocking of S1PR1, S1PR2, or S1PR3 could antagonize the phosphorylation of ERK1/2, S1P receptors antagonists were applied to human BMSCs. S1PR1 antagonist W146 and S1PR3 antagonist CAY10444 treatment completely abrogated S1P-mediated ERK1/2 phosphorylation, whereas S1PR2 antagonist JTE-013 did not exert a similar effect (Figure 5(e)).

Taken together, these data indicate that S1P induces human BMSC migration through a G_*i*_/ERK-dependent pathway via S1PR1 and S1PR3.

### 3.6. Rho/Rho-Associated Protein Kinase (ROCK) Pathway Participates in S1PR2-Mediated Inhibition of Human BMSC Migration

**Figure 6:**
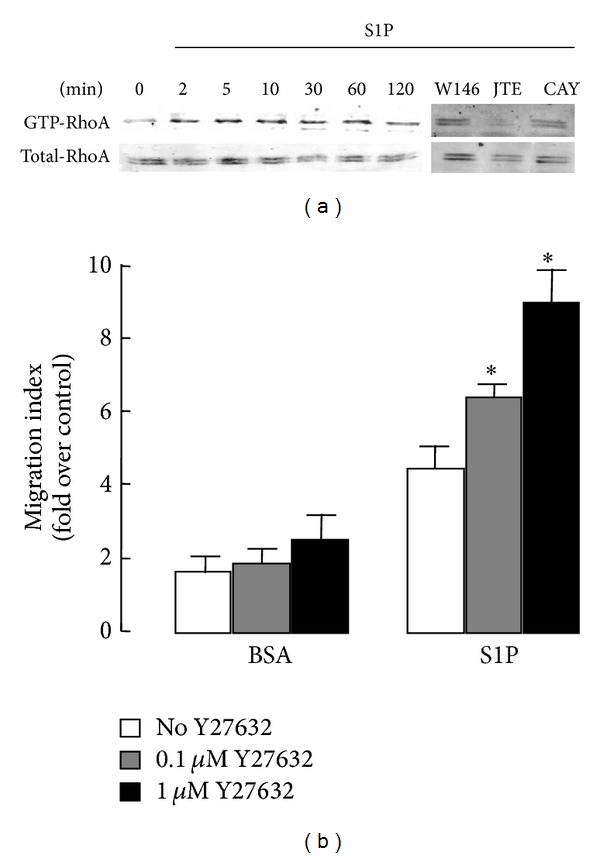
The essential role of the Rho pathway in S1PR2-mediated suppression of human BMSCs migration. (a) Left: cells were stimulated with S1P (1 *μ*M) for the indicated time. Right: cells were pretreated with S1PRs antagonists (S1PR1 antagonist W146, S1PR2 antagonist JTE-013, and S1PR3 antagonist CAY1444) and then were stimulated with 1 *μ*M S1P for 5 min. RhoA activities were determined by a pull-down assay. (b) Serum-starved BMSCs were pretreated with the indicated concentration of Y27632 and then were allowed to migrate for 4 h in the presence of 1 nM S1P.

Pull-down assay showed that RhoA was rapidly and consistently activated by S1P (Figure 6(a)). It has been shown that activated GTP-bound Rho activates several downstream signaling pathways, among which ROCK is a prominent player [28]. Therefore, we tested the effects of a ROCK inhibitor, Y27632, on BMSC migration and found that cells treated with Y27632 were considerably augmented in S1P-induced migration of BMSCs (Figure 6(b)). Taking our previous data involving S1PR2 into consideration, we further investigated whether S1PR2, which couples to G_12/13_, correlated with the activation of RhoA. As Figure 6(a) shows, S1PR2 antagonist JTE-013 treatment of cells completely blocked S1P-mediated activation of Rho, whereas S1PR1 antagonist W146 and S1PR3 antagonist CAY10444 did not have such an effect. These data suggest that S1P activates Rho via S1PR2 and that downstream ROCK activity is required for the inhibition of migration.

## 4. Discussion

S1P is a bioactive lipid released by many cells during inflammation and following injury which can regulate different cellular functions [16]. Moreover, interaction between S1P and its five specific G protein-coupled receptors is known to regulate many physiological and pathophysiological processes including cancer, inflammation, diabetes, and several immune disorders [18]. One of the profound activities of extracellular S1P is to mediate cell migration, which is the focus of this study. S1P-triggered migration of MSCs to sites of injured tissues is required for cell therapy and tissue reconstitution. In the present study, we described a potent effect regulated by the S1P/S1PRs axis which has implications for the initiation of immunotherapy or tissue regeneration. Our results suggest that S1PR1 and S1PR3 play an important role in S1P-induced migration of human BMSCs, whereas S1PR2 negatively regulates S1P-induced migration in human BMSCs. Furthermore, G_*i*_-dependent activation of ERK1/2 as well as the Rho/ROCK pathway is involved in this process.

Along with previous studies in other cell types, S1P was demonstrated to serve as a good candidate for the induction of human BMSC motility, and S1PR1–3 were shown to be specifically involved in this action [29–34]. By employing pharmacologic tools and RNA interference technology, we further identified S1PR1/3-mediated stimulatory and S1PR2-mediated inhibitory signaling in S1P-induced migration of human BMSCs, which was similar to the phenomenon in LX-2 cells [35] and human myofibroblasts [36]. However, conflicting results were found in studies on human lung fibroblasts which indicated that S1P potentiates fibroblast chemotaxis through S1PR2 [37]. There are some possible explanations for these contradictory results. First, the gelatin migration assay which was used in the human lung fibroblast studies shows an invasive effect, but not chemotaxis activity. Thus, it is unsurprising that the invasion assay displayed opposite results to the transwell assay by our group and others. In addition, S1PR2 was reported to decrease glioma cell motility but enhance invasion through inducing cell interaction with the extracellular matrix and matrix degradation in glioma cell [38]. Therefore, S1PR2-mediated interaction with matrix might play an important role in these contradictory studies. With regards to the role of S1PR2 in invasion versus chemotaxis, more detailed mechanisms should be studied.

The varied cellular effects of S1P were mainly attributed to the coupling of these receptors to different G proteins and their differential signaling cascades. In particular, S1P receptor coupling to G_*i*_ leads to cell migration, while coupling to G_12/13_ leads to inhibition of cell migration [24]. In this study, S1P-induced migration of BMSCs via S1PR1 and S1PR3 was observed to be sensitive to PTX, thereby implicating G_*i*_-linked signaling pathways. Indeed, the S1PR1 is known to couple to G_*i*_, whereas S1PR2 and S1PR3 couple to G_*i*_, G_*q*_, and G_12/13_ [24, 39]. S1PR1 is a G_*i*_-coupled receptor that stimulates migration in mouse embryonic fibroblasts [33] and lymphocytes [34, 40]. In contrast, S1PR2, coupled more strongly to G_12/13_, is known to inhibit migration in other cell types, like vascular smooth muscle cells [41] and glioma cells [38]. However, which G proteins are involved in S1PR3-mediated cell motility remained unknown. As discussed, S1PR3 exhibited stimulatory effects in human myofibroblast [36] and LX-2 cells [35] but had no effects in lymphocytes [34, 40]. Since S1PR3 couples to members of the G_*i*_, G_*q*_, and G_12/13_ families, such differences are probably attributed to the varying affinities for the subsets of G proteins found in different cell types. In the present work, we observed that S1PR3 stimulated migration as well as S1PR1, which implies that S1PR3 coupled more effectively to G_*i*_ than G_*q*_, and G_12/13_ in human BMSCs. Thus, a further insight into the mechanistic details of S1P/S1PRs/G protein signaling axis is necessary to understand human BMSCs migration.

The MAPK/ERK pathway has been widely demonstrated to play an essential role in cell survival, proliferation, and migration [25, 26, 42]. Indeed, S1P-mediated cell motility was regulated by the MAPK signal transduction pathway in different cell types. In particular, ERK1/2 activation was often associated with an S1PR1-G_*i*_-dependent pathway [26, 43, 44]. Additionally, S1PR2 and S1PR3 were also observed to induce rapid and reversible S1P-mediated ERK phosphorylation [45–48]. However, which S1P receptor subtype leads to activation of ERK and cell migration in human BMSCs remained unknown. Here, we found that both S1PR1 and S1PR3, but not S1PR2, were required for activation of ERK and contributed to chemotaxis towards S1P in human BMSCs. Our finding that ERK activation is a result of S1P signaling through S1PR1 and S1PR3 is in agreement with previous studies in other cells. For example, NADPH oxidase activity and intracellular H_2_O_2_ levels increase in NIH3T3 fibroblasts as a result of activated ERK caused by S1P signaling [46]. However, there are also reports of S1PR3 exerting no effects on ERK1/2 activation, but instead inducing the activation of Akt [44]. Furthermore, S1PR2, a receptor often exhibiting inhibitory effects on migration, can lead to the activation of ERK1/2 in primary rat hepatocytes [45]. The reason for such discrepancies is not clear yet, although different cell types used in these studies may be one of the causes.

In addition, S1PR2 coupling with G_12/13_ was demonstrated to result in activation of Rho with subsequent inhibition of cell motility. In accordance with previous findings, our study suggested that Rho/ROCK activity was required for inhibiting S1P-induced migration in human BMSCs. In contrast, gelatin migration assay used in some previous studies exhibited the activation of Rho induced migration in endothelial cells and bone marrow cells [19, 49]. Meriane et al. have implied that matrix metalloproteinase-mediated migration stimulated by S1P in the gelatin migration assay was associated with an increase in Rho activity and actin stress fibers [19], whereas the transwell assay displayed enhanced chemotactic migration corresponding to reduced Rho activity and stress fibers in bone marrow stromal cells [50]. As S1P-triggered Rho activation is mainly mediated by S1PR2, the role for Rho in migration is probably corresponding to S1PR2 [24]. Similar to that S1PR2 exerts opposite migration effects, Rho-mediated stimulatory invasive activity, and inhibitory chemotactic effect. In particular, mediation of stress fibers may confound the interpretation of these experiments. Interestingly, during chemotaxis, an increase in stress fiber formation might not be required when S1P is acting as a chemoattractant. In contrast, actin stress fiber formation is necessary in invasive migration which involves matrix degradation [19, 50]. Therefore, migration mediated by S1P is a complicated process which requires a different activation status of the actin cytoskeleton, including gradient sensing, polarization, and orientation versus subsequent migration at different stages of activation. Further studies are expected to elucidate more mechanistic details involved in the varied migration of human BMSCs.

In summary, our present study demonstrates that activation of S1PR1 or S1PR3 increases migration of human BMSCs by activating of MAPK/ERK pathway via G_*i*_ protein. In contrast, activation of S1PR2 decreases migration via Rho activation. These results suggest that the coupling of S1P receptors to various heterotrimeric G proteins and, consequently, distinct downstream signaling pathways lead to downstream pathological phenomena. It is noted that S1PR1/3 and S1PR2 as well as the MAPK/ERK and Rho/ROCK pathways help to balance migration of human BMSCs mediated by S1P. Importantly, our results unravel an important part of the steps that lead to the activation of directional migration in BMSCs, and they may allow us to better develop BMSCs as an improved cellular therapy for immunological disorders or tissue regeneration.
